# Graft and patient outcomes after abdominal normothermic regional perfusion in liver transplantation

**DOI:** 10.1308/rcsann.2026.0076

**Published:** 2026-07-23

**Authors:** S Komperla, K Chall, R Seeruthun, B Clay

**Affiliations:** ^1^ East Suffolk and North Essex NHS Foundation Trust, UK; ^2^ University Hospitals Brimingham NHS Foundation Trust, UK; ^3^ Western Health and Social Care Trust, UK; ^4^ East Suffolk and North Essex NHS Foundation Trust, UK

**Keywords:** Liver transplantation, aNRP, DCD

## Abstract

**Introduction:**

Donation after circulatory death (DCD) liver transplantation is currently limited by inferior outcomes compared with donation after brain death (DBD), predominantly due to ischaemia–reperfusion injury and biliary complications. Abdominal normothermic regional perfusion (aNRP) is an emerging graft-preservation technique that aims to improve graft viability, patient outcomes and reduce rate of complications in DCD liver transplantation. The aim of this review is to appraise the current literature on the utility of aNRP in liver transplantation surgery.

**Methods:**

The PRISMA guidelines for systematic reviews were followed and relevant databases searched. Primary outcomes were graft and patient survival rates. Secondary outcomes were primary non-function, early allograft dysfunction, biliary complications including ischaemic cholangiopathy (IC)/ischaemic type biliary lesions (ITBL) and retransplantation rates. Risk-of-bias analysis was conducted. Narrative synthesis was performed due to marked heterogeneity in the included studies.

**Results:**

Fourteen studies (2014–2026) encompassing 1,278 transplanted aNRP liver grafts were included in this review. aNRP was associated with a reduction in IC/ITBL rates, lower rates of primary non-function and lower early allograft dysfunction compared with conventional retrieval (super rapid recovery followed by static cold storage). Graft loss and retransplantation rates were also lower in aNRP cohorts. Compared with DBD transplantation, aNRP outcomes were similar with near-zero rates of IC in both groups. No significant differences were also observed between aNRP and ex situ normothermic machine perfusion (NMP).

**Conclusions:**

aNRP is associated with reductions in biliary complications, graft loss and retransplantation compared with conventional DCD retrieval, with outcomes comparable to DBD and ex situ NMP and offers a solution to expand the donor pool.

## Introduction

Globally there is a growing gap between the demand for liver transplantation and the availability of suitable donor grafts. In The United Kingdom (UK), 1,617 patients were listed for liver transplantation in 2020 yet only 767 patients received a transplant; 599 patients remained on the waiting list and 68 patients died waiting.^1^ This shortage has driven efforts to expand the potential donor pool and improve utilisation of marginal grafts, including grafts from donation after circulatory death (DCD) donors. DCD is the donation of organs after death has been declared following irreversible cardiopulmonary arrest. DCD donors can be classified according to the Maastricht Classification.^2^

Although donation after brain death (DBD) remains the preferred benchmark for deceased donor liver transplantation, DCD grafts are being increasingly utilised to expand the potential donor pool with their utility increasing significantly in the past decade.^3^ However, DCD liver transplantation has historically been associated with inferior outcomes compared with DBD transplantation, including lower graft survival rates, higher rates of primary non-function (PNF), early allograft dysfunction (EAD), biliary complications and increased recipient mortality.^4–6^ These poorer outcomes can be attributed to the warm ischaemic injury sustained after withdrawal of life-sustaining treatment and before graft perfusion. In addition, standard procurement and preservation techniques that are effective in DBD transplantation, particularly super rapid recovery (SRR) followed by static cold storage (SCS) are less effective in DCD grafts because they cannot reverse the metabolic consequences of warm ischaemic injury.^7^ This problem is further exacerbated by the fact that the biliary tree is particularly susceptible to ischaemia–reperfusion injury in DCD liver transplantation, which contributes to the development of ischaemic cholangiopathy (IC), ischaemic-type biliary lesions (ITBL) and other non-anastomotic biliary complications.^8^

Abdominal normothermic regional perfusion (aNRP) is an in situ graft reperfusion technique used in DCD donation after death has been confirmed and the required no-touch period has elapsed.^9^ By restoring oxygenated blood perfusion to the abdominal organs before retrieval, aNRP may partially reverse the metabolic consequences of warm ischaemia and reduce ischaemia–reperfusion injury while also allowing for functional assessment of donor graft viability prior to transplantation.^9,10^ In addition to aNRP, other graft-preservation strategies such as ex situ normothermic machine perfusion (NMP) and hypothermic oxygenated machine perfusion have also emerged and their role in DCD liver transplantation is increasingly being studied.^11^

Despite increasing utility in clinical practice, the current evidence base for aNRP in DCD liver transplantation remains heterogeneous, with studies variably comparing aNRP-assisted DCD transplantation with conventional non-aNRP DCD pathways, with DBD transplantation or with machine perfusion techniques. The aim of this systematic review was to evaluate the current clinical evidence for aNRP in DCD liver transplantation with particular focus on comparative outcomes vs non-aNRP DCD pathways and benchmark outcomes relative to DBD transplantation.

## Methods

The review was conducted in accordance with the Preferred Reporting Items for Systematic Reviews and Meta-Analyses (PRISMA) guidelines (http://www.prisma-statement.org/). A study protocol was developed and registered on PROSPERO (CRD42024501263).

### Inclusion and exclusion criteria

Randomised controlled trials, cohort studies and case-controlled studies evaluating the use of aNRP in DCD liver transplantation were included. Studies that compared aNRP DCD cohorts against non-aNRP DCD cohorts, DBD cohorts or NMP cohorts were included. Inclusion was limited to studies conducted on human participants and written in English only. Case reports, letters to the editor, editorials, systematic reviews, conference abstracts, unpublished data and non-human studies were excluded. In the case of repeated/duplicate data sets, the most recently published study was included.

### Search strategy

A comprehensive literature search of the following electronic databases was conducted: PubMed, Ovid Embase, Scopus and Cochrane Library. An initial review of the literature was conducted to identify alternative keywords and Medical Subject Heading terms to include in our search strategy. The search was initially run to identify all eligible studies published from January 1972 to January 2024. A re-run of the search was conducted to retrieve newer studies published between January 2024 and January 2026. The full search strategy can be found in Supplementary Material 1.

The records retrieved were transferred to EndNote X7 reference manager software for screening.^12^ All duplicates were removed prior to title and abstract screening, which was conducted by two authors (SK, KC) in accordance with the inclusion and exclusion criteria, with any disagreements resolved through discussion with a third reviewer (RS). Studies that were eligible from the title and abstract screening underwent full-text screening by two reviewers (SK, KC), with disagreements resolved by discussion prior to data extraction. An exclusion reason was recorded for all excluded studies during full-text review.

### Data extraction

All studies included following full-text screening were transferred to a separate spreadsheet and data extraction was performed using a predefined template. Extracted variables included study characteristics, donor and recipient characteristics, aNRP protocol details, graft and patient survival, PNF, EAD, biliary complications including IC/non-anastomotic biliary lesions and retransplantation rates where reported. Post-aNRP preservation techniques were also recorded where available.

### Risk-of-bias evaluation

The methodological quality of the studies was assessed using the Newcastle–Ottawa quality assessment tool.^13^ Any discrepancies were discussed between the authors.

### Data analysis

A meta-analysis was considered but not performed because of significant clinical and methodological heterogeneity across the included studies. Studies differed in comparator groups, donor selection thresholds and post-normothermic regional perfusion (NRP) preservation techniques. In addition, outcome reporting was also variable with differing use of terms (IC vs ITBL vs non-anastomotic stricture [NAS]) and non-uniform time point reporting of survival outcomes. Finally, some national/multicentre cohorts may have represented overlapping populations. As a result, narrative synthesis was considered most appropriate.

## Results

Fourteen studies met the inclusion criteria and were included in the final review.^14–27^ Figure 1 shows the PRIMSA flow diagram for study screening and selection.

**Figure 1:**
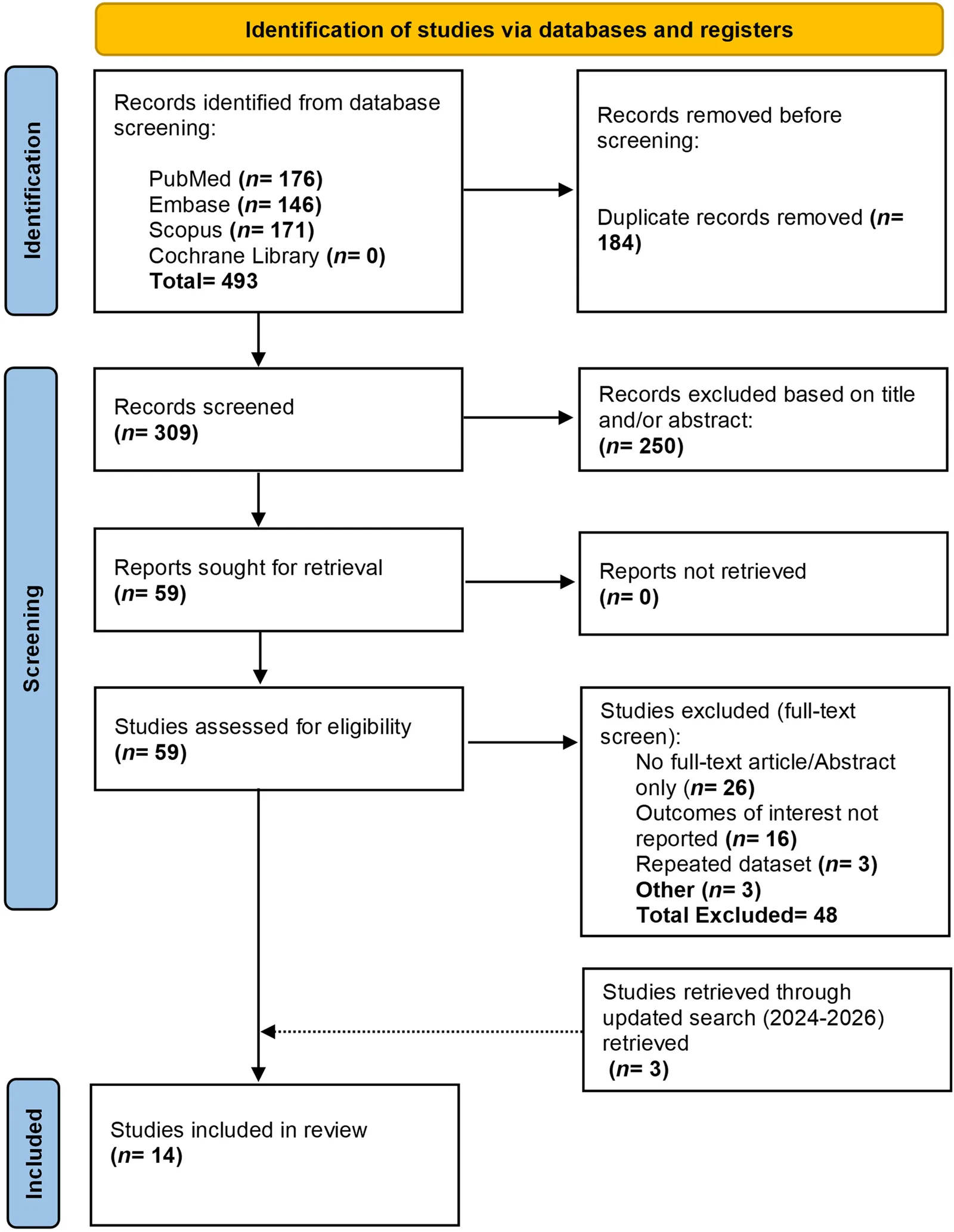
PRISMA flowchart

### Study characteristics

Table 1 shows the characteristics of the included studies. All 14 studies were observational in design and originated from the UK, the United States, Spain, Italy, France, Sweden or an international collaborative group.^25^ Most of the studies compared aNRP in controlled DCD liver transplantation, although two studies included mixed abdominal and thoracoabdominal regional perfusion in their patient cohorts.^14,16^ Five studies compared aNRP with SCS (with or without SRR) in DCD transplants, six studies compared aNRP DCD transplantation with DBD transplantation, and one study compared aNRP with NMP in DCD transplantation.^14–25^ Gaurav *et al* was a three-arm study comparing aNRP with both SRR followed by SCS and NMP.^18^ Two studies were single-arm aNRP cohorts with no comparator group.^26,27^ Across the 14 included studies, 1,278 transplanted aNRP liver grafts were reported with a mean aNRP donor age of approximately 51 years. This reflects the transplanted cohort only and not the total number of potential donors because many of the studies had a larger pool of potential donor grafts that were not transplanted or were discarded owing to viability reasons. Donor graft type (Maastricht Classification), donor selection criteria and donor characteristics are shown in Table 1.

**Table 1:** Summary of study characteristics

Study	Study design	Location	Study period	aNRP group (*n*)	aNRP donor type (Maastricht)	Comparator group (*n*)	Comparator donor type	Donor selection criteria
Watson *et al* 2019^14^	Retrospective two-centre cohort study	UK	January 2011–June 2017	43	cDCD (III)	187	Non-NRP cDCD with SCS	No cirrhotic/severely steatotic livers; ALT >200IU/l declined
Hessheimer *et al* 2022^15^	Retrospective multicentre cohort study	Spain	2012–2019	545	cDCD (III)	258	cDCD with SRR+SCS	fWIT <30min
Brubaker *et al* 2024^16^	Retrospective multicentre cohort study	USA	January 2017–May 2023	106	cDCD (III)	136	cDCD with SRR+SCS	Centre-specific cDCD donor thresholds
Croome *et al* 2025^17^	Retrospective single-centre cohort study	USA	2022–2024	83 (62 aNRP alone; 21 aNRP+NMP)	cDCD (III)	297	cDCD with SCS	Programme-specific viability and WIT thresholds
Gaurav *et al* 2022^18^	Retrospective single-centre three-arm cohort study^*^	UK	January 2013–October 2020	69	cDCD (III/IV)	97 (SCS arm) 67 (NMP arm)	cDCD with SRR+SCS; cDCD with ex situ NMP	UK programme-specific thresholds
Ruiz *et al* 2021^19^	Retrospective single-centre cohort study	Spain	January 2015–June 2019	100	cDCD (III)	200	DBD	fWIT <30min; ALT <3× upper normal limit
Rodriguez *et al* 2022^20^	Retrospective single-centre cohort study	Spain	November 2016–April 2021	39	cDCD (III)	78	DBD	—
Miñambres *et al* 2020^21^	Retrospective two-centre cohort study	Spain	September 2014–December 2018	16	cDCD (III)	29	DBD	ALT/AST ≤4× upper normal limit during aNRP
Savier *et al* 2020^22^	Retrospective multicentre cohort study	France	February 2015–December 2019	50	cDCD (III)	100	DBD	Age <55; CIT <8h; ALT <200IU/l
Bluhme *et al* 2024^23^	Prospective national pilot study with matched historical controls	Sweden	2020–2022	18	cDCD	28	DBD	Age ≤65; BMI ≤28; Agonal period ≤60min; fWIT ≤30min; CIT <8h; no macrosteatosis
De Carlis *et al* 2018^24^	Retrospective cohort study	Italy	2015–2017	20	cDCD (III)/uDCD (II)	52	DBD	Age ≤65; WIT ≤160min; ALT ≤1,000IU/l; downward trend in serum lactate
Mohkam *et al* 2022^25^	Retrospective international multicentre cohort study	International	August 2014–December 2019	34	cDCD	68	cDCD with ex situ NMP	Age 18–75; CIT <9h
Antoine *et al* 2020^26^	Prospective single-arm cohort study	France	January 2015–December 2018	123	cDCD (III)	—	—	Age <66; fWIT <30min
Oniscu *et al* 2014^27^	Prospective pilot study	UK	—	11	cDCD (III)	—	—	fWIT <30min; ALT <4× upper normal limit at end of aNRP

ALT = alanine aminotransferase; aNRP = abdominal normothermic regional perfusion; BMI = body mass index; cDCD = controlled donation after circulatory death; CIT = cold ischaemia time; DBD = donation after brain death; fWIT = functional warm ischaemia time; NMP = normothermic machine perfusion; SCS = static cold storage; SRR = super rapid recovery; uDCD = uncontrolled DCD

*Gaurav *et al* was a three-arm study with two comparator groups: SCS (*n* = 97) and ex situ NMP (*n*=67).

### aNRP characteristics

Table 2 shows the aNRP characteristics and post-aNRP preservation techniques utilised. Most studies evaluated aNRP followed by SCS. De Carlis *et al* utilised hypothermic machine perfusion (HMP) following aNRP in all 20 DCD liver grafts.^24^ Croome *et al* included a distinct aNRP + NMP sequential subgroup.^17^ No-touch periods were usually 5-min except for De Carlis *et al* which had a 20-min no-touch period as per Italian laws.^24^ Thresholds and definitions for warm ischaemia time (WIT), functional warm ischaemia time (fWIT), cold ischaemia time (CIT) and aNRP duration varied significantly between studies.

**Table 2:** aNRP characteristics and post-aNRP preservation techniques utilised^*^

Study	Median/mean WIT (range/IQR)	Median/mean fWIT (range/IQR)	Median/mean aNRP duration (range/IQR)	Temperature target (°C)	aNRP flow rate (l/min)	Median/mean CIT (range/IQR)	Ex situ graft preservation technique
Watson *et al* 2019^14^	NR	—	123min (103–130)	—	2.5–3	382min (303–502)	SCS
Hessheimer *et al* 2022^15^	18min (13–23)	12min (9–16)	111min (81–126)	35–37	2.2–2.4	320min (270–379)	SCS
Brubaker *et al* 2024^16^	22min (18–25)	—	62min (51–89)	35–37	2.0–5.0	289min (218–355)	SCS
Croome *et al* 2025^17^	26.4±12.2 min (aNRP alone); 38.8±26.6 min (aNRP+NMP)	—	67min (35–263) aNRP alone; 72min (32–202) aNRP+NMP	—	—	6.2±1.6h (aNRP alone); 13.4±3.3h (aNRP+NMP)	SCS or sequential NMP
Gaurav *et al* 2022^18^	29min (23–33)	19min (15–24)	133min (121–143)	—	—	399min (341–471)	SCS
Ruiz *et al* 2021^19^	—	10min (8.5–12.2)	121min (118–128)	—	—	274min (241–311)	SCS
Rodriguez *et al* 2022^20^	19.23min (10–38)	13.19min (6–26)	90–120min	—	—	298.21min	SCS
Miñambres *et al* 2020^21^	16min (12–19)	12min (10–13)	114min (58–121)	37	2.0–2.24	311min	SCS
Savier *et al* 2020^22^	—	22min (20–226.8)	190min (151–223)	—	—	348min (300–402)	SCS
Bluhme *et al* 2024^23^	28.8min (mean)	—	124min (mean)	—	—	336min (sd ±70.8)	SCS
De Carlis *et al* 2018^24^	125min (72–143)	—	352min (308–434)	—	—	8h (IQR 6–9)	HMP†
Mohkam *et al* 2022^25^	31min (27–37)	21min (18–25)	184min	—	—	346min (285–397)	SCS
Antoine *et al* 2020^26^	—	22min (18–26)	186min (161–207)	—	—	341min (279–392)	SCS
Oniscu *et al* 2014^27^	16min (10–23)	26min (13–48)	120min	35.5–37.5	1.7–4.0	389min (169–450)	SCS

aNRP = abdominal normothermic regional perfusion; CIT = cold ischaemia time; fWIT = functional warm ischaemia time; HMP = hypothermic machine perfusion; IQR = interquartile range; NMP = normothermic machine perfusion; NRP = normothermic regional perfusion; SCS = static cold storage; WIT = total warm ischaemia time

*No-touch periods were 5min in all centres except De Carlis *et al* (Italy, legally mandated 20min)

†De Carlis *et al*: all 20 donation after circulatory death grafts received HMP (LiverAssist, OrganAssist) following aNRP0 outcomes reflect the combined aNRP+HMP pathway, not aNRP alone

### Primary outcomes

#### aNRP vs SCS with or without SRR in DCD transplantation

Table 3 shows graft and patient survival rates across the studies. Five studies had aNRP cohorts compared with SCS with or without SRR (SCS±SRR) in DCD transplantation.^14–18^ Overall, graft-related outcomes favoured aNRP over SCS±SRR, with three studies^15,17,18^ demonstrating statistically significant superior graft survival in aNRP cohorts and one study demonstrating higher graft survival rates that did not reach statistical significance.^14^ Brubaker *et al* reported no explicit numerical survival data but found comparable graft and patient survival between both NRP and SCS±SRR cohorts.^16^ Four studies showed higher patient survival rates with aNRP compared with SCS±SRR, although only Croome *et al* demonstrated statistical significance.^14,15,17,18^ Studies reported a combination of 90 day, 1-year and 3-year survival rates, with some reporting 2- and 5-year outcomes, reflecting the wide heterogeneity in follow-up duration and reporting.

**Table 3:** Primary outcome data from included studies (12 of 14 studies reported primary outcome data^*^).

Study	Cohort	Graft survival at 90 days (95% CI)	Graft survival at 1 year (95% CI)	Graft survival at 3 years (95% CI)	Patient survival at 90 days (95% CI)	Patient survival at 1 year (95% CI)	Patient survival at 3 years (95% CI)
Watson *et al* 2019^14^	aNRP (*n* = 43)	97.7% (84.6–99.7)	—	—	100.0%	—	—
	cDCD with SCS (*n* = 187)	89.8% (84.5–93.4)	—	—	97.3% (93.7–98.9)	—	—
	*p*	0.10	—	—	0.28	—	—
Hessheimer *et al* 2022^15^	aNRP (*n* = 540)	—	90%	87%	—	92%	89%
		cDCD with SRR (*n* = 257)	—	79%	68%	—	86%	76%
		*p*	—	—	HR 0.435 (0.313–0.604) *p* < 0.001	—	—	HR 0.517 (0.357–748); *p*<0.001
	Croome *et al* 2025^17^	aNRP alone (*n* = 62)	98.3%	96.5%	96.5%	100%	98.2%	98.2%
		aNRP+NMP (*n* = 21)	100%	100%	—	100%	100%	—
		cDCD with SCS (*n* = 297)	91.6%	87.5%	83.7%	97.3%	94.0%	90.3%
		*p* (aNRP alone vs SCS)	0.016	—	—	—	—	0.16
	Gaurav *et al* 2022^18^	aNRP (*n* = 69)	—	97%	94%	—	94%	94%
		NMP (*n* = 67)	—	91%	82%	—	94%	90%
			cDCD with SCS (*n* = 97)	—	88%	86%	—	94%	88%
			*p* (aNRP vs SCS)	—	—	HR 0.4 (0.2–0.8) *p* = 0.034	—	—	0.665
		Ruiz *et al* 2021^19^	aNRP (*n* = 100)	—	99%	93%	—	99%	94%
			DBD (*n* = 200)	—	92%	87%	—	96%	90%
			*p*	—	0.04	0.28	—	–	0.07
		Rodriguez *et al* 2022^20^	aNRP (*n* = 39)	—	—	—	—	94%	71%
			DBD (*n* = 78)	—	—	—	—	84%	75%
			*p*	—	—	—	—	—	—
		Miñambres *et al* 2020^21^	aNRP (*n* = 16)	—	—	—	87.5%	87.5%	—
			DBD (*n* = 29)	—	—	—	96%	96%	—
			*p*	—	—	—	0.50	0.50	—
		Savier *et al* 2020^22^	aNRP (*n* = 50)	98%	88%	—	98%	90%	—
			DBD (*n* = 100)	95%	85%	—	99%	88%	—
			*p*	0.66	0.91	—	0.8	0.68	—
		Bluhme *et al* 2024^23^	aNRP (*n* = 18)	—	94.4%	—	—	94.4%	—
			DBD (*n* = 28)	—	96.4%	—	—	100%	—
			*p*	—	0.60	—	—	–	—
		De Carlis *et al* 2018^24^	aNRP (*n* = 20)	—	85% (60–95%)	—	—	95% (69–99%)	—
			DBD (*n* = 52)	—	91% (80–97%)	—	—	94% (82–98%)	—
			*p*	—	0.20	—	—	0.94	—
		Mohkam *et al* 2022^25^	aNRP (*n* = 34)	—	93.9%	89.4%	—	98.5%	96.4%
			ex situ NMP (*n* = 68)	—	88.2%	88.2%	—	94.1%	90.9%
				*p*	—	HR 0.67 (0.20–2.23) *p* = 0.516	—	—	—	HR 0.38 (0.06–2.30) *p* = 0.275
			Antoine *et al* 2020^26^ (single arm)	aNRP (*n* = 123)	93.1% (85.9–96.6%)	89.7% (81.5–94.3%)	—	—	93.6% (86.3–97.1%)	—

aNRP = abdominal normothermic regional perfusion; cDCD = controlled donation after circulatory death; CI = confidence interval; DBD = donation after brain death; GS = graft survival; HR = hazard ratio; NMP = normothermic machine perfusion; PS = patient survival; SCS = static cold storage; SRR = super rapid recovery

*Brubaker *et al* reported comparable graft and patient survival between cohorts with no explicit numerical figures; Oniscu *et al* did not have formal numerical outcomes

#### aNRP vs DBD transplantation

Six studies compared outcomes from aNRP DCD transplantation with DBD transplantation (Table 3).^19–24^ Graft and patient survival rates were generally comparable between aNRP DCD transplantation and DBD transplantation. Ruiz *et al* was the only study to reach statistical significance for overall graft survival with the aNRP DCD group achieving superior 1- and 3-year graft survival compared with DBD (99% and 93% vs 92% and 87%; *p* = 0.04).^19^ No study demonstrated a significant difference in patient survival rates between aNRP DCD and DBD cohorts. Across the six studies, a total of 243 aNRP DCD transplants were compared against 487 DBD transplants.

#### aNRP vs NMP in DCD transplantation

Two studies evaluated aNRP against ex situ NMP (Table 3).^18,25^ Gaurav *et al* conducted a three-arm study that included an NMP as well as an SCS comparator group and found that both aNRP and NMP resulted in improved early graft function relative to SCS, but aNRP was associated with the lowest retransplantation rate (4% vs 12% vs 18%; *p* = 0.04), lowest overall biliary complications (22% vs 37% vs 42%; *p* = 0.024) and lowest incidence of clinically significant NAS (0% vs 11% vs 14%; *p* = 0.009).^18^ Mokham *et al* found similar 1- and 2-year graft and patient survival rates between aNRP and NMP cohorts.^25^

#### Single-arm aNRP studies

Two studies evaluated aNRP outcomes without a comparator cohort. Antoine *et al* reported 1-year graft and patient survival rates of 89.7% and 93.6%, respectively, in a large French cohort of 123 aNRP DCD transplantations (Table 3).^26^ The study also found that graft survival was significantly lower in grafts whose transplantation deviated from the national protocol compared with those in which it was strictly adhered to, highlighting the importance of standardised evidence-based protocols (68.4% vs 94.8%; *p* < 0.01). Oniscu *et al* demonstrated multicentre feasibility in an early UK DCD liver transplantation cohort (*n* = 11), although explicit numerical outcomes were incompletely reported.^27^

### Secondary outcomes

#### aNRP vs SCS±SRR in DCD transplantation

Table 4 shows the secondary outcomes reported by the studies. Biliary outcomes consistently favoured aNRP across all five studies that evaluated it against SCS±SRR.^14–18^ Notably, the incidence of IC/ITBL/NAS (terminology varied between studies) was lower in aNRP cohorts across all five studies, with three studies reporting no cases of IC or NAS in their aNRP cohorts.^14,16,17^ Furthermore EAD and PNF were also lower with aNRP across all five studies, with four studies reporting no cases of PNF in the aNRP groups. Retransplantation rates also favoured aNRP, with Hessheimer *et al* (6 of 545 [1%] vs 24 of 258 [9%]; odds ratio [OR] 0.26, *p* < 0.001) and Gaurav *et al* (3 of 69 [4%] vs 17 of 97 [18%]; *p* = 0.04) reporting significantly lower rates with aNRP compared with SCS±SRR.^15,18^

**Table 4:** Secondary outcome data from included studies.

Study	Cohort	PNF *n*/*N* (%)	EAD *n*/*N* (%)	Biliary complications (any) *n*/*N* (%)	IC/ITBL/NAS *n*/*N* (%)	Retransplantation *n*/*N* (%)
Watson *et al* 2019^14^	aNRP (*n* = 43)	0/43 (0)	5/43 (12)	3/43 (7)	0/42 (0)	—
	cDCD with SCS (*n* = 187)	4/187 (13 overall graft losses)	55/173 (32%)	18/174 (10%)	47/171 (27%)	—
	*p*	—	0.008	0.77	< 0.0001	—
Hessheimer *et al* 2022^15^	aNRP (*n* = 540)	16/540 (3)	81/540 (15)	65/540 (12)	∼5/540 (1)	6/540 (1)
		cDCD with SRR (*n* = 257)	15/257 (6)	59/257 (23)	75/257 (29)	∼23/257 (9)	24/258 (9)
		*p*	—	OR 0.56 (0.36–0.87) *p* = 0.010	OR 0.32 (0.21–0.49) *p* < 0.001	OR 0.12 (0.04–0.30) *p* < 0.001	OR 0.26 (0.14–0.49) *p* < 0.001
	Brubaker *et al* 2024^16^	aNRP (*n* = 106)	0/106 (0)	77/106 (36.4)	7/105 (6.7)	0/104 (0)	—
		cDCD with SRR (*n* = 136)	2/136 (1.5%)	123/136 (56.1%)	30/134 (22.4%)	12/133 (9.0%)	—
		*p*	0.21	0.007	0.001	0.002	—
	Croome *et al* 2025^17^	aNRP alone (*n* = 62)	0/62 (0)	7/62 (11.3)	—	0/62 (0)	3/62 (4.8)
		aNRP+NMP (*n* = 21)	0/21 (0)	9/21 (42.9)	—	0/21 (0)	—
		cDCD with SCS (*n* = 297)	6/297 (2.0)	187/297 (64.5)	—	50/297 (16.8)	—
		*p* (aNRP alone vs SCS)	0.26	*p* < 0.001	—	*p* < 0.001	—
	Gaurav *et al* 2022^18^	aNRP (*n* = 69)	0/69 (0)	10/69 (14)	—	0/69 (0)	3/69 (4)
		NMP (*n* = 67)	—	—	—	7/67 (10.4)	8/67 (12)
		cDCD with SCS (*n* = 97)	5/97 (5.2)	35/97 (36)	—	22/97 (22.7)	17/97 (18)
		*p* (aNRP vs SCS)	—	0.008	—	HR 0.3 (0.1–0.6) *p* = 0.005	0.04
	Ruiz *et al* 2021^19^	aNRP (*n* = 100)	0/100 (0)	21/100 (21.0)	5/100 (5.2)	0/100 (0)	1/100 (1.0)
		DBD (*n* = 200)	—	38/200 (19.2)	13/200 (6.3)	0/200 (0)	7/200 (3.5)
		*p*	—	—	0.90	—	0.29
	Rodriguez *et al* 2022^20^	aNRP (*n* = 39)	0/39 (0)	—	5/39 (12.0)	0/39 (0)	0/39 (0)
		DBD (*n* = 78)	3/78 (3.8)	—	4/78 (5.1)	1/78 (1.3)	8/78 (10.3)
		*p*	—	—	0.104	—	—
	Miñambres *et al* 2020^21^	aNRP (*n* = 16)	2/16 (12.5)	3/16 (18.8)	—	0/16 (0)	1/16 (6.3)
		DBD (*n* = 29)	0/29 (0)	5/29 (17.2)	—	0/29 (0)	1/29 (3.4)
		*p*	0.12	0.60	—	—	0.59
	Savier *et al* 2020^22^	aNRP (*n* = 50)	—	9/50 (18.0)	8/50 (16.0)	—	0/50 (0)
		DBD (*n* = 100)	—	32/100 (32.0)	17/100 (17.0)	—	1/100 (1.0)
		*p*	—	0.11	0.94	—	—
	Bluhme *et al* 2024^23^	aNRP (*n* = 18)	0/18 (0%)	4/18 (22.2)	—	0/18 (0)	—
		DBD (*n* = 28)	1/28 (3.6)	8/28 (28.6)	—	2/28 (7.1)	—
		*p*	—	—	—	—	—
	De Carlis *et al* 2018^24^	aNRP (*n* = 20)	2/20 (10.0)	4/17 (23.5)	4/20 (20.0)	2/20 (10.0)	3/20 (15.0)
		DBD (*n* = 52)	2/52 (3.8)	13/49 (26.5)	7/52 (13.5)	2/52 (3.8)	3/52 (5.8)
		*p*	0.58	> 0.99	0.65	0.52	0.35
	Mohkam *et al* 2022^25^	aNRP (*n* = 34)	1/34 (1.5)	7/34 (20.6)	8/34 (11.8)	1/34 (1.5)	—
		ex situ NMP (*n* = 68)	1/68 (2.9%)	6/68 (8.8)	7/68 (20.6)	2/68 (2.9)	—
		*p*	1.00	0.13	0.249	> 0.99	—
	Antoine *et al* 2020^26^ (single arm)	aNRP (*n* = 123)	3/123 (2.4)	4/123 (3.3)	—	1/123 (0.8)	1/123 (0.8)
	Oniscu *et al* 2014^27^ (single arm)	aNRP (*n* = 11)	1/11 (9.1)	4/11 (36.4)	2/11 (18.2)	0/11 (0)	—

aNRP = abdominal normothermic regional perfusion; BC = biliary complications (any type); cDCD = controlled donation after circulatory death; DBD = donation after brain death; EAD = early allograft dysfunction (Olthoff criteria unless otherwise stated); HR = hazard ratio; IC = ischaemic cholangiopathy; ITBL = ischaemic-type biliary lesions; NAS = non-anastomotic strictures; NMP = normothermic machine perfusion; OR = odds ratio; PNF = primary non-function; SCS = static cold storage; SRR = super rapid recovery

#### aNRP vs DBD transplantation

Across the six studies comparing aNRP with DBD transplantation, rates of biliary complication, IC/ITBL/NAS, EAD, PNF and retransplantation were generally comparable between both cohorts, although none of the reported results reached statistical significance (Table 4).^19–24^ Ruiz *et al* and Bluhme *et al* reported lower rates across most secondary outcomes with aNRP in DCD transplantation compared with DBD transplantation, with no cases of ITBL or NAS in aNRP cohorts.^19,23^ De Carlis *et al* reported a higher rate of IC (10%) and retransplantation (15%) in their aNRP DCD cohort compared with matched DBD cohorts, although it is important to note that the prolonged WIT (median 125min ) as a result of Italy’s 20-min no-touch period and the sequential use of aNRP and HMP may have a significant confounding role.^24^

#### aNRP vs NMP in DCD transplantation

Gaurav *et al* demonstrated that both aNRP and NMP resulted in not only superior outcomes compared with SCS, but also lower clinically significant NAS rates (0 of 66 vs 7 of 63 [11%]; *p* = 0.009) and fewer retransplantations (3 of 69 [4%] vs 8 of 67 [12%]; *p* = 0.04) with aNRP compared with ex situ NMP (Table 4).^18^ Mokham *et al* reported low rates of biliary complications and symptomatic NAS that were similar in both aNRP and NMP cohort, with no significant difference in any outcomes.^25^

#### Single-arm aNRP studies

Antoine *et al* reported rates of PNF (3 of 123 [2.4%]) and EAD (4 of 123 [3.3%]) in a large aNRP DCD cohort with a single case of ITBL occurring in a recipient with a fWIT exceeding 30min owing to protocol deviation (Table 4).^26^ Oniscu *et al* reported no ITBL in 11 recipients but reported a high rate of EAD (4 of 11 [36.4%]), which was higher than other studies in this review but may reflect the early learning phase of aNRP utilisation and absence of strict donor selection criteria.^27^

### Risk-of-bias analysis

Risk-of-bias analysis was conducted for all 14 studies using the Newcastle–Ottawa Scale for cohort studies.^13^ Seven studies were graded as moderate risk and seven were graded as low risk. The main sources of bias were retrospective observational study design, limited follow-up reporting and incomplete adjustment for baseline differences between cohorts. The full risk-of-bias analysis is shown in Table 5.

**Table 5:** Risk-of-bias analysis using Newcastle–Ottawa Scale

Study	Newcastle–Ottawa Scale									
	Selection				Comparability	Outcome			Total score (out of 9)	Risk-of-bias grade
	Representativeness of exposed cohort	Selection of non-exposed cohort from same source as exposed cohort	Ascertainment of exposure	Outcome of interest not present at start of study	Comparability of cohorts	Assessment of outcome	Follow-up long enough for outcome to occur	Adequacy of follow-up		
Watson *et al* 2019^14^	Somewhat representative of the average in the community ★	Drawn from the same community as the exposed cohort ★	Secure record (surgical records) ★	Yes ★	Controlled for same donor type (cDCD) ★	Record linkage (eg identified through ICD codes on database records) ★	Yes ★	No description/unclear	7	Low
Hessheimer *et al* 2021^15^	Somewhat representative of the average in the community ★	Drawn from the same community as the exposed cohort ★	Secure record (surgical records) ★	Yes ★	Controlled for same donor type (cDCD) ★ ★	Record linkage (eg identified through ICD codes on database records) ★	Yes ★	No description/unclear	8	Low
Brubaker *et al* 2024^16^	Somewhat representative of the average in the community ★	Drawn from the same community as the exposed cohort ★	Secure record (surgical records) ★	Yes ★	Controlled for same donor type (cDCD) ★	Record linkage/secure database records ★	Yes ★	No description/unclear	7	Low
Croome *et al* 2025^17^	Somewhat representative of the average in the community ★	Drawn from the same community as the exposed cohort ★	Secure record (surgical records) ★	Yes ★	Controlled for same donor type/pathway comparison ★	Record linkage/secure database records ★	Yes ★	No description/unclear	7	Low
Gaurav *et al* 2022^18^	Somewhat representative of the average in the community ★	Drawn from the same community as the exposed cohort ★	Secure record (surgical records) ★	Yes ★	Controlled for same donor type (cDCD) ★	Record linkage/secure database records ★	Yes ★	No description/unclear	7	Low
Ruiz *et al* 2021^19^	Somewhat representative of the average in the community ★	Drawn from a different source ★	Secure record (surgical records) ★	Yes ★	Controlled through matched design/recipient comparison ★★	Record linkage (eg identified through ICD codes on database records) ★	Yes ★	No description/unclear	8	Low
Rodriguez *et al* 2022^20^	Somewhat representative of the average in the community ★	Drawn from a different source ★	Secure record (surgical records) ★	Yes ★	Cohorts not controlled for confounding factors	Record linkage (eg identified through ICD codes on database records) ★	Yes ★	No description/unclear	6	Moderate
Miñambres *et al* 2020^21^	Somewhat representative of the average in the community ★	Drawn from a different source ★	Secure record (surgical records) ★	Yes ★	Cohorts not controlled for confounding factors	Record linkage (eg identified through ICD codes on database records) ★	Yes ★	No description/unclear	6	Moderate
Savier *et al* 2020^22^	Somewhat representative of the average in the community ★	Drawn from a different source ★	Secure record (surgical records) ★	Yes ★	Cohorts not controlled for confounding factors	Record linkage (eg identified through ICD codes on database records) ★	Yes ★	No description/unclear	6	Moderate
Bluhme *et al* 2024^23^	Somewhat representative of the average in the community ★	Drawn from a different source ★	Secure record (surgical records) ★	Yes ★	Controlled through matched design ★★	Record linkage/secure database records ★	Yes ★	No description/unclear	8	Low
De Carlis *et al* 2018^24^	Somewhat representative of the average in the community ★	Drawn from a different source ★	Secure record (surgical records) ★	Yes ★	Controlled through matching design ★★	Record linkage (eg identified through ICD codes on database records) ★	Yes ★	No description/unclear	8	Low
Mohkam *et al* 2022^25^	Somewhat representative of the average in the community ★	Drawn from a different source ★	Secure record (surgical records) ★	Yes ★	Controlled through propensity-matched design ★★	Record linkage/secure database records ★	Yes ★	No description/unclear	8	Low
Antoine *et al* 2020^26^	Somewhat representative of the average in the community ★	No non-exposed cohort involved	Secure record (surgical records) ★	Yes ★	No control	Record linkage (eg identified through ICD codes on database records) ★	Yes ★	No description/unclear	5	Moderate
Oniscu *et al* 2014^27^	Somewhat representative of the average in the community ★	No non-exposed cohort involved	Secure record (surgical records) ★	Yes ★	No control	Record linkage (eg identified through ICD codes on database records) ★	Yes ★	No description/unclear	5	Moderate

cDCD = controlled donation after circulatory death; ICD = International Classification of Diseases. Each star (★) represents one point on the Newcastle–Ottawa Scale, with a maximum of nine (four for Selection, two for Comparability, three for Outcome). Higher scores indicate lower risk of bias; studies scoring ≥ 7 were graded low risk, 5–6 moderate risk and ≤ 4 stars as high risk. Single-arm studies (Antoine *et al*, Oniscu *et al*) were ineligible for stars in the comparability domain and for selection of a non-exposed cohort.

## Discussion

This review synthesises the most up-to-date evidence on aNRP in DCD liver transplantation and demonstrates that aNRP generally shows superior graft, patient, biliary and retransplantation outcomes compared with SCS±SRR. Furthermore, when compared with DBD transplantation and NMP DCD transplantation, aNRP demonstrates comparable survival and biliary outcomes, suggesting a valuable role as an in situ graft-preservation technique.

When comparing aNRP with SCS±SRR in DCD transplantation, lower rates of IC, ITBL, NAS, EAD and retransplantation were generally observed with aNRP. Interestingly although graft survival also favoured aNRP, patient survival differences were less consistent and only one study demonstrated a statistically significant survival benefit.^15^ This is a key point to highlight because the major barriers in DCD liver transplantation have been the higher rates of EAD, IC and avoidable graft loss, and not solely survival rates.^28^ The results of this review suggest that aNRP can improve graft quality and viability even if an obvious survival benefit is not demonstratable. Furthermore, the fact that these outcomes consistently favoured aNRP across different countries and centres supports the idea that these results are due to a biological advantage of aNRP rather than a region-specific trend.

The biological basis for aNRP in DCD transplantation has been studied both clinically and in laboratory testing. Unlike DBD grafts, DCD grafts are exposed to a mandatory period of warm ischaemia after circulatory arrest and the no-touch interval, resulting in toxic metabolite build-up.^29^ Unlike conventional SCS, aNRP reperfuses the graft with warm oxygenated blood potentially allowing for partial mitochondrial functional recovery, replenishment of hepatocyte energy stores and washout of accumulated metabolites. The benefit of aNRP is particularly relevant to the biliary tree, which is disproportionately susceptible to ischaemia–reperfusion injuries and underpins the major complications and hurdles currently seen in DCD transplantation with biliary complications.^8^

Another important observation highlighted by our review is that DCD liver transplantation using aNRP can achieve outcomes comparable with DBD transplantation. Across the six studies comparing aNRP DCD transplantation with DBD transplantation, graft and patient survival rates were generally comparable and no study demonstrated a statistically significant difference in patient survival. It is important to note that this does not imply that DCD and DBD grafts are biologically equivalent but rather that aNRP may help make DCD grafts ‘good enough' to enable wider clinical utility.

Our review also synthesises data from two studies comparing aNRP with advanced machine-assisted perfusion strategies like NMP.^18,25^ Both studies included in our review reported comparable-to-favourable outcomes with aNRP in comparison with NMP. Furthermore, Croome *et al* had a subgroup of grafts that received sequential aNRP and NMP and reported 100% graft and patient survival rates at 90 days and 1 year.^17^ These findings suggest that the next step in aNRP evaluation is comparative studies not only against other advanced perfusion strategies, but also comparing different sequential strategies to achieve best possible outcomes.

Another finding this review highlights are the substantial variations in aNRP protocols, donor selection criteria and post-aNRP preservation techniques. No-touch periods ranged from 5min in most centres to 20min in Italy resulting in substantially longer warm ischaemia times. Furthermore, the donor selection criteria varied significantly between studies, and between studies in the same country, with differing warm ischaemia thresholds and viability assessments; the importance of this is evident, with Antonine *et al* reporting significantly lower survival rates in grafts that did not follow national protocol.^26^

The findings of this study should be interpreted in the context of prior reviews. Van de Leemkolk *et al* provided an early evaluation of aNRP in DCD transplantation but encompassed all abdominal organs rather than liver transplantation specifically and was less able to report liver-specific outcomes.^30^ More recently, Mastrovangelis *et al* performed a systematic review and meta-analysis of aNRP in DCD liver transplantation and found similar findings to the current review with improved outcomes relative to non-aNRP DCD and comparable outcomes with DBD transplantation.^31^ However, the review predated several newer studies and did not fully address post-aNRP preservation techniques or make comparisons with ex situ NMP.

### Study limitations

Several limitations of the evidence base warrant acknowledgement. All 14 studies were observational, with the majority retrospective, leaving findings vulnerable to selection bias. Outcome reporting was also heterogeneous across studies, particularly for biliary outcomes, which consisted of differing terminologies and diagnostic criteria, limiting direct cross-study comparison. Graft and patient survival outcomes were also reported with inconsistent time points, preventing robust meta-analysis.

## Conclusions

In conclusion, aNRP demonstrates consistently superior graft outcomes, lower rates of biliary complications and fewer overall retransplantations compared with conventional DCD procurement techniques while also achieving comparable outcomes with DBD transplantation ex situ NMP. Future research should be focused on developing optimal aNRP-based preservation pathways, exploring the role of different sequential perfusion strategies and standardising protocol and donor selection criteria.

## Competing interests

The author/s declare no competing interests.

## Funding

The author/s received no financial support for the research, authorship and/or publication of this article.

## Ethics approval and consent to participate

This study is a systematic review of previously published data and did not involve direct human participants, human tissue, or identifiable patient information requiring ethical approval. .

## Author contributions

**S Komperla:** Conceptualisation, Data curation, Formal analysis, Investigation, Methodology, Validation, Visualisation, Writing – original draft, Writing – review & editing. **K Chall:** Data curation, Formal analysis, Investigation, Methodology, Writing – review & editing. **R Seeruthun:** Methodology, Writing – review & editing. **BJ Clay:** Data curation, Formal analysis, Visualisation, Writing – review & editing.

## Artificial Intelligence

The author/s declare that no AI was used to conduct the study or prepare the manuscript.
